# Systematic Review and Meta-analysis: Association of Aspirin With Incidence of Hepatocellular Carcinoma

**DOI:** 10.3389/fphar.2022.764854

**Published:** 2022-03-01

**Authors:** Xueliang Zhou, Tengfei Zhang, Yali Sun, Chunwei Li, Xianfei Ding, Yanhui Zhu, Lifeng Li, Zhirui Fan

**Affiliations:** ^1^ Department of Interventional Radiology, The First Affiliated Hospital of Zhengzhou University, Zhengzhou, China; ^2^ Cancer Centre, The First Affiliated Hospital of Zhengzhou University, Zhengzhou, China; ^3^ General ICU, The First Affiliated Hospital of Zhengzhou University, Henan Key Laboratory of Critical Care Medicine, Zhengzhou, China; ^4^ Internet Medical and System Applications of National Engineering Laboratory, Zhengzhou, China; ^5^ Integrated Traditional and Western Medicine, The First Affiliated Hospital of Zhengzhou University, Zhengzhou, China

**Keywords:** hepatocellular carcinoma, aspirin, HCC, meta-analysis, systematic review 3

## Abstract

**Aim:** To explore the relationship between the use of aspirin and the incidence of hepatocellular carcinoma (HCC).

**Methods:** MEDLINE, EMBASE, Web of Science and Cochrane CENTRAL databases were searched systematically from the earliest available date to 13 March 2020. The primary outcome was incidence of HCC, and the secondary outcomes were recurrence and mortality of HCC. The results were expressed as the Hazard Ratio (HR) and 95% confidence interval (CI). Based on the heterogeneity evaluated with the *I* ^*2*^ statistic, a meta-analysis was performed using either a random- or fixed-effects model.

**Results:** A total of sixteen articles (2781100 participants) were included. There was lower incidence of HCC in aspirin users than those in non-aspirin users (HR, 0.56; 95% CI, 0.46-0.69; *p* < 0.001). Subgroup analysis further showed that the incidence of liver cancer in patients with alcoholic cirrhosis (HR, 0.14; 95% CI, 0.09-0.22; *p* < 0.001) and virus hepatitis (HR, 0.68; 95% CI, 0.62-0.74; *p* < 0.001) who use aspirin was lower than that of patients who do not use aspirin. In addition, aspirin was found to associate with decreased risk of HCC mortality (HR, 0.71; 95% CI, 0.65-0.78; *p* < 0.001), not HCC recurrence (HR, 0.52; 95% CI, 0.15-1.76; *p* = 0.291).

**Conclusions:** Aspirin use is significantly associated with the low incidence rate of liver cancer.

## 1 Introduction

Hepatocellular carcinoma (HCC) accounts for a large proportion of cancer deaths worldwide ((Torre et al., 2015; Bray et al., 2018)), and the incidence of HCC is predicted to increase in the future ((Torre et al., 2015; Bray et al., 2018)). HCC can grow at an exponential rate; its recurrence can occur after a therapy and its subsequent metastasis can lead to mortality, making it the second cause of death of cancer patients ((Torre et al., 2015; Bray et al., 2018)). The current diagnosis of HCC remains ineffective; thus, it is important that preventive methods are developed ((Torre et al., 2015; Bray et al., 2018)). The main risk factors for HCC are chronic hepatitis and virus infection, in particular hepatitis B virus (HBV) and hepatitis C virus (HCV), and long-term drinking ((Chen et al., 1991; Degos et al., 2000; Yeh et al., 2010)). Several studies have suggested that chronic hepatitis inflammation could induce HCC((Raza et al., 2011; Hossain et al., 2012; Li et al., 2013a)), especially occurring through the cyclooxygenase-2 (COX-2) pathway ((Cheng et al., 2004)). Thus, modulation of the inflammatory pathways may become a novel method that can restrict HCC development.

Aspirin is a COX inhibitor frequently used to reduce the risk of cardiovascular and cerebrovascular disease-related death, owing to its antiplatelet effect. Moreover, aspirin has been shown to play a role in preventing lung cancer, colorectal cancer and prostate cancer ((Cao et al., 2016; Lapi et al., 2016; Ye et al., 2019)). It has been also shown to have a beneficial effect in liver, such as lowering the risk of hepatic inflammation, fibrosis, and HCC, according to the data from chronic hepatitis animal model ((Cao et al., 2016; Lapi et al., 2016; Ye et al., 2019)). The mechanisms underlying the chemopreventive effect of aspirin, however, remain unknown. Recent studies have explored the associations between HCC and the chemopreventive effect of aspirin that is associated with chronic inflammation. Beside the inflammatory process ((Sitia et al., 2012; Carrat 2014)), aspirin has been suggested to modulate immune response of liver and promote the liver injury mediated by immune and carcinogenesis ((Semple et al., 2011)). This information demonstrates that aspirin plays roles in pathogenesis of HCC.

Given the evidence of aspirin’s chemopreventive effect, it may potentially be prevent HCC. Recent articles have indicated that due to anti-inflammation and immune modulation effect of aspirin ((Sahasrabuddhe et al., 2012; Petrick et al., 2015; Hwang et al., 2018)), number of HCC incidence in patients taking aspirin is lower compared with that in patients without aspirin. Conversely, several studies have reported that use of aspirin has no notable effect on the incidence of HCC ((Sahasrabuddhe et al., 2012; Petrick et al., 2015; Hwang et al., 2018)). Given such equivocal information, it is necessary to compile and evaluate data from available articles to determine whether aspirin is beneficial for preventing the incidence of HCC.

## 2 Methods

### 2.1 Search Strategy

The meta-analysis was performed based on the Meta-analysis of Observational Studies in Epidemiology guidelines and the protocol of this study in accordance with the Preferred Reporting Items for Systematic Reviews and Meta-analysis (PRISMA) checklist ((Stroup et al., 2000)). And the PRISMA checklist is shown in Supplementary Table S1. Articles written in English language published from the earliest possible date to 13 March 2020 in the MEDLINE, EMBASE, Web of Science and Cochrane CENTRAL databases and were searched using a combination of MeSH/Emtree and title/abstract keywords. The keywords were “Acetylsalicylic acid,” “Aspirin,” “Hepatocellular Carcinoma,” “Liver cancer,” “Hepatic cellular cancer,” and “HCC”. Supplementary Table S2 showed the detailed search strategy.

### 2.2 Inclusion and Exclusion Criteria

#### 2.2.1 Incidence

The inclusion criteria of this study are as follows (Torre et al., 2015): they enrolled patients using aspirin and non-aspirin for prevention or treatment (Bray et al., 2018); they counted the HCC incidence of aspirin and non-aspirin users (McGlynn and London 2011); they were adults patients (age ≥18 years) (Altekruse et al., 2012); they were observational studies or clinical trials (Villanueva 2019); they were written in English. The articles were excluded when (Torre et al., 2015): These studies lacked the results of the correlation between the use of aspirin and HCC incidence rate and hazard ratio (HR), relative risk, odd ratio (OR) and 95% confidence interval (CI), or did not provide raw data (Bray et al., 2018).; they were reviews, commentaries, editorials, conference abstracts, or animal studies (McGlynn and London 2011); they assessed the impact of aspirin combined with other NSAIDs on the incidence of HCC.

#### 2.2.2 Recurrence and Mortality

The inclusion criteria of this study are as follows (Torre et al., 2015): they enrolled HCC patients who used aspirin and non-aspirin (Bray et al., 2018); they counted the HCC recurrence, mortality of patients using aspirin and non-aspirin (McGlynn and London 2011); they were adults patients (age ≥18 years) (Altekruse et al., 2012); they were observational studies or clinical trials (Villanueva 2019); they were written in English. The articles were excluded when (Torre et al., 2015): the studies lacked outcome data for correlation between the use of aspirin and HCC recurrence, mortality with hazard ratio (HR), relative risk, or odd ratio(OR) value and 95% confidence interval (CI), or not provide the raw data as we can calculate out the result (Bray et al., 2018); they were reviews, commentaries, editorials, conference abstracts, or animal studies (McGlynn and London 2011); they assessed the effect of aspirin in combination with other NSAIDs on the recurrence, mortality of HCC.

### 2.3 Inclusion of Studies and Data Extraction

These articles were first extracted based on the inclusion criteria by two investigators; after that, the differences between them were determined by another investigator. The articles that were included into this study contain the following information: first author’s name, year of publication, area where the study was conducted, study design, study period, total number of aspirin/no-aspirin users, HCC number of aspirin/no-aspirin users, liver disease status, primary and secondary outcomes, definition of aspirin user, aspirin dose, adjusted variables. We show these in Table 1 and Table 2. Aspirin users was defined as people who had used aspirin before or after HCC, and the specific information was shown in Table 2. If available, the HR and its related 95% CI were extracted directly from the original article. If not, the HR and 95% CI were calculated according to the raw data in the study. In addition, several case-control studies of this meta-analysis only provide the odd ratio not HR ((Yang et al., 2016; Shen et al., 2020)), so we treated the odd ratio is approximately equivalent to the HR value and then to pooled together because the incidence of HCC is far below 5% (Akinyemiju et al., 2017), namely if research results are rare in all subjects and subgroups, people can usually ignore the differences between various relative risk measures (such as odds ratio, ratio and risk ratio) (Greenland 1987; Siristatidis et al., 2013; Grant 2014).

**TABLE 1:** Baseline characteristics for studies included in the meta-analysis.

Study	Region	Study design	Study period	Total number of aspirin/no-aspirin users	HCC number of aspirin/no-aspirin users	Liver disease status	Reason for aspirin	Primary outcome (HR, 95% CI)	Secondary outcome (HR, 95% CI)
								Incidence	Recurrence	Mortality
Jacobs et al. (2012)^41^	America	RC	1997–2018	23869/76270	22/15	NA	NA	NA	NA	0.52 (0.30–0.93)
Sahasrabuddhe et al. (2012)^21^	America	PC	1995–2008	219291/81213	159/90	NA	NA	0.59 (0.45–0.77)	NA	NA
Yeh et al. (2014)^44^	Taiwan	RC	01/1997-12/2010	377/15197	9812/5762	Curative liver resection	NA	NA	0.82 (0.64–1.06)	NA
Petrick et al. (2015)^20^	America	RC	1985–2010	477470/606663	368/313	NA	NA	0.68 (0.57–0.81)	NA	NA
Li et al. (2016)^42^	Chinese (Mainland)	RC	01/2008-12/2013	60/60	60/60	unresectable HCC	treatment of cardiovascular disease, transient ischemic attack, and arthritis	NA	NA	0.498 (0.28–0.888)
Yang et al. (2016)^22^	Britain	RCC	1988–2011	1670/4165	376/819	NA	NA	1.11 (0.86–1.44)	NA	NA
Hwang et al. (2018)^19^	Korea	RC	01/2007-12/2013	64782/395973	382/1954	NA	NA	0.87 (0.77–0.98)	NA	NA
Simon et al. (2018)^36^	America	PC	1980–2012	58855/74516	NA	NA	Headache, musculoskeletal pain and primary cardiovascular disease prevention (NHS), and cardiovascular disease risk reduction, pain, and headache (HPFS)	0.51 (0.34–0.77)	NA	NA
Du et al. (2019)^40^	Chinese (Mainland)	RC	01/2000-12/2014	59/205	NA	HBV, HCV, cirrhotic, after splenectomy	Postoperative long-term low-dose aspirin administration	0.16 (0.04–0.88)	NA	0.281 (0.049–0.96)
Lee et al. (2019)^34^	Taiwan	RC	01/1997-12/2012	2123/8492	NA	HBV	antiplatelet therapy for cardiovascular diseases	0.71 (0.58–0.86)	NA	NA
Tsoi et al. (2019)^35^	Chinese (Mainland)	RC	2000–2013	204170/408339	1984/7386	NA	to prevent cardiovascular and cerebrovascular diseases	0.49 (0.45–0.53)	NA	NA
Young et al. (2019)^43^	Taiwan	RC	10/2007-05/2014	15/415	15/32	HBV, curative resection of HCC	coronary artery disease, type 2 diabetes mellitus or before the surgery of HCC	NA	0.221 (0.054–0.915)	0.582 (0.143–2.365)
Liao et al. (2020)^37^	Taiwan	RC	2000–2012	1911/1911	131/147	HCV	treated with aspirin	0.56 (0.43–0.72)	NA	NA
Shen et al. (2020)^24^	America	RCC	01/2011-02/2016	676/1129	186/466	NA	NA	0.39 (0.30–0.52)	NA	NA
Shin et al. (2020)^38^	Korea	RC	08/2003-05/2016	224/725	NA	alcoholic cirrhosis	aspirin therapy	0.14 (0.09–0.22)	NA	NA
Simon et al. (2020)^39^	Sweden	RCC	07/2005-12/2013	14205/36070	338/1274	HBV, HCV	cardiovascular prevention	0.69 (0.62–0.76)	NA	0.73 (0.67–0.81)

PC, prospective cohort; RC, retrospective cohort; RCC, retrospective case-control; HCC, hepatocellular carcinoma; HBV, hepatitis B virus; HCV, hepatitis C virus; HR, hazard ratio; CI, confidence interval; NA, not available.

**TABLE 2:** Definition of aspirin user, aspirin dose and adjusted variables.

Study	Definition of aspirin user	Aspirin dose	Adjusted variables
Jacobs et al. (2012)^41^	Use 30 or 31 days per month of either low-dose or adult-strength aspirin	Low-dose or adult-dose aspirin	Age, sex, race, education, smoking, history of heart disease, stroke, diabetes, hypertension, cholesterol-lowering drug use (current), aspirin use in the year 1982, nonsteroidal anti-inflammatory drug use, and history of colorectal endoscopy (ever)
Sahasrabuddhe et al. (2012)^21^	Self-reported aspirin use	Monthly (≤2–3 times per month), weekly (1–2 times to 5–6 times per week), or daily use (≥1 times per day)	Age, sex, race, cigarette smoking, alcohol consumption, diabetes, and body mass index
Yeh et al. (2014)^44^	Retrieved from the pharmacy register data set	NA	Age, sex, extent of liver resection, chronic viral hepatitis status, comorbidities, and the use of drugs such as statin and metformin
Petrick et al. (2015)^20^	Get from ten US-based prospective cohort studies	NA	Age, sex, race, cohort, body mass index, smoking, drinking, diabetes
Li et al. (2016)^42^	Administered at least 100 mg/day of aspirin continuously for more than 3 months	≥100 mg/day	Age, gender, date of HCC diagnosis, Child-Pugh score, following treatment after the initial TACE, tumor size, tumor number, vascular invasion, and metastasis the initial date of HCC diagnosis
Yang et al. (2016)^22^	Having two or more aspirin prescriptions recorded prior to the index date of the individual	NA	Body mass index, smoking status, alcohol-related disorders, hepatitis B or C virus infection, diabetes, rare metabolic disorders, and use of paracetamol, antidiabetic medications, and statins
Hwang et al. (2018)^19^	Used more than 365 DDDs of aspirin	≥365 DDDs	Age, sex, body mass index, health behaviors (cigarette smoking, alcohol consumption, and physical activity), concurrent medication, category of blood pressure, fasting plasma glucose and total cholesterol, socioeconomic status, and Charlson comorbidity index score
Simon et al. (2018)^36^	≥standard-dose [325-mg] tablets per week	≥325 mg/week	Body mass index, alcohol intake, smoking status, physical activity, diabetes, hypertension, dyslipidemia, Regular multivitamin use, regular use of oral antidiabetic medications, regular use of statins, regular use of non-aspirin nonsteroidal anti-inflammatory drugs
Du et al. (2019)^40^	Taking 100 mg/d aspirin within 7 days	100 mg/d	Gender, AST, INR, surgical method, postoperative early aspirin
Lee et al. (2019)^34^	Received daily aspirin for 90 or more days	≤100 mg/d	Age, male sex, liver cirrhosis, diabetes, hyperlipidemia, hypertension, statin use, metformin use, and Nucleoside analogues use
Tsoi et al. (2019)^35^	Adults with aspirin prescription for at least 6 months	The median dose of aspirin was 80 mg	Age, sex
Young et al. (2019)^43^	Continuous use of aspirin for at least 30 days before tumor recurrence	NA	Age, sex, and other covariates
Liao et al. (2020)^37^	NA	NA	Age, sex, comorbidities, drugs, diagnosis year, and index year
Shen et al. (2020)^24^	At least once per week over a duration of 3 months or more	NA	Age, gender, race, education, household income, and marital status
Shin et al. (2020)^38^	Who were treated with aspirin more than 6 months	100 mg/day	NA
Simon et al. (2020)^39^	Identified by their first filled prescriptions for 90 or more consecutive doses of aspirin	Low-dose aspirin (≤160 mg)	Sex; continuous years since diagnosis of hepatitis B or hepatitis C; liver disease severity, hypertension, obesity, or alcohol abuse or misuse; and use of insulin, metformin, and statins, and so on

US, United States; HCC, hepatocellular carcinoma; AST, aspartate aminotransferase; INR, international normalized ratio; DDD, defined daily dose; NA, not available.

### 2.4 Assessment of Risk of Bias

We included thirteen cohort studies and three case-control studies in this meta-analysis. The Newcastle Ottawa scale (NOS) was used to assess the risk of bias for each outcome in all included studies ((BS et al., 2021)). According to the study population selection, comparability, and adequacy of the outcome data, a total of nine points were obtained articles with seven to nine points are considered high-quality articles ((Yang et al., 2016; Shen et al., 2020)) Publication bias was assessed for the primary outcome only. The result of the NOS was shown in Supplementary Table S3.

### 2.5 Primary and Secondary Outcomes

The main study outcome of this meta-analysis was incidence of HCC, and the secondary outcomes were recurrence and mortality of HCC.

### 2.6 Statistical Analysis

Heterogeneity between articles was presented as HRs and 95% CIs, and were determined using a random or fixed effect model. Heterogeneity was further assessed by Chi^2^ and *I* ^2^ test, in which the percentage of variability was determined (not sampling error) (Higgins and Thompson 2002; Higgins et al., 2003). Articles with *p* values <0.10 or *I* ^2^ value >50% was considered having substantial heterogeneity. If there is heterogeneity, subgroup analysis will be conducted to explore the potential sources of heterogeneity and consider whether the random effects model can be used for meta-analysis. In addition, Begg funnel plot and Egger’s linear regression were used to evaluate the potential publication bias of the main results of the included studies ((Begg and Mazumdar 1994; Stuck et al., 1998)). Dissymmetry was evaluated visually by Funnel plots. For Egger’s tests, *p* < 0.1 indicated a significantly small study size. The robustness of the results for primary outcome was evaluated by one-way sensitivity analysis. All statistical analyses were conducted using STATA 14.0 (College Station, Texas 77845 United States, Serial number: 401406267051). Differences for which *p* < 0.05 (two-sided) were considered statistically significant.

## 3 Results

### 3.1 Included Studies

**FIGURE 1:**
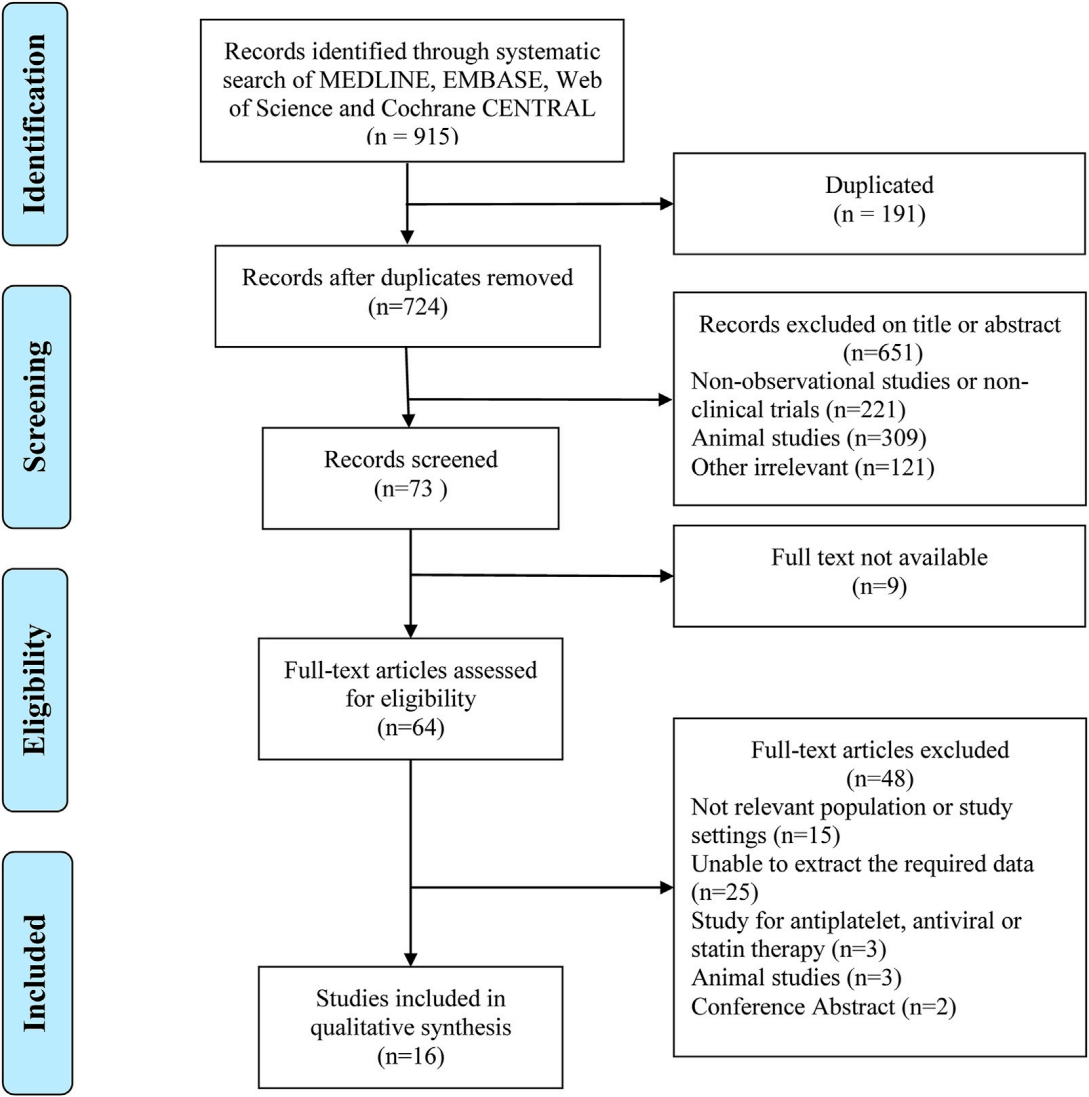
Workflow for database search used in the meta-analysis.

A total of 915 relevant articles were searched, among which 191 articles were duplicates and were removed. After the initial search, 64 articles that discuss problems related to the main goal of this study were selected. Finally, 16 articles ((Sahasrabuddhe et al., 2012; Petrick et al., 2015; Yang et al., 2016; Hwang et al., 2018), (Sahasrabuddhe et al., 2012; Petrick et al., 2015; Yang et al., 2016; Hwang et al., 2018), (Jacobs et al., 2012; Yeh et al., 2015; Li et al., 2016; Simon et al., 2018; Du et al., 2019; Lee et al., 2019; Tsoi et al., 2019; Liao et al., 2020; Shin et al., 2020; Simon et al., 2020; Young et al., 2020)), in which 2781100 patients were involved, and selected (Figure 1).

### 3.2 Characteristics of Included Studies

The included articles were observational studies that reported the relationship between aspirin and HCC. Among which, twelve articles reported the relationship between aspirin and incidence of HCC((Sahasrabuddhe et al., 2012; Petrick et al., 2015; Yang et al., 2016; Hwang et al., 2018), (Sahasrabuddhe et al., 2012; Petrick et al., 2015; Yang et al., 2016; Hwang et al., 2018), (Simon et al., 2018; Du et al., 2019; Lee et al., 2019; Tsoi et al., 2019; Liao et al., 2020; Shin et al., 2020; Simon et al., 2020)), two articles showed the relation between aspirin and recurrence of HCC((Yeh et al., 2015; Young et al., 2020)), and five articles indicated the connection between aspirin and mortality of HCC((Jacobs et al., 2012; Li et al., 2016; Du et al., 2019; Simon et al., 2020; Young et al., 2020)). All the included articles gained NOS of greater than or equal to 7 points, suggesting that there is a low risk of bias. The characteristics of the included articles are shown as Table 1 and Table 2.

### 3.3 Primary and Secondary Outcome

**FIGURE 2:**
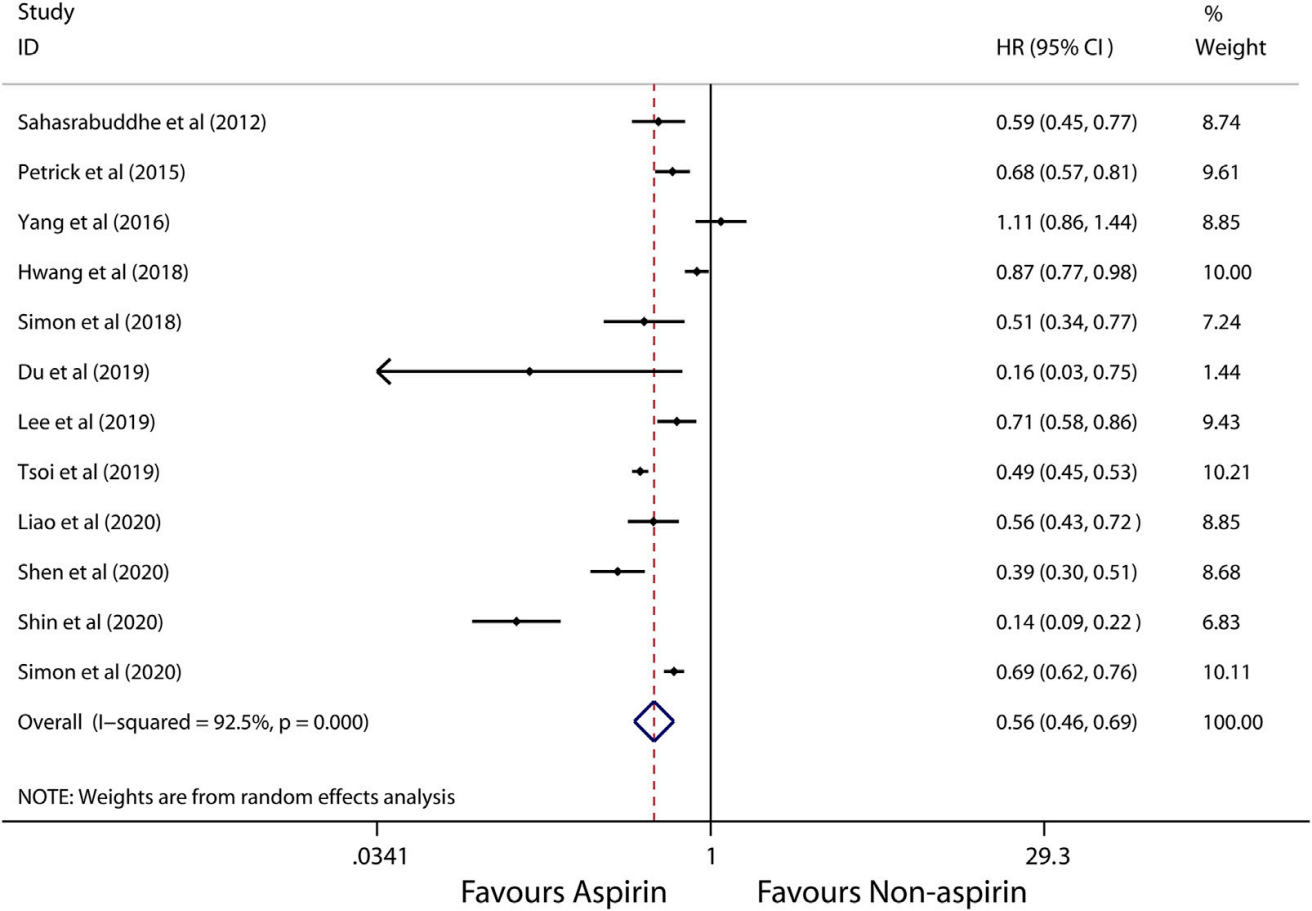
Meta-analysis of overall pooled HRs with 95% CIs across studies for primary outcomes. Forest plot showing the significance of the relationship between aspirin use and incidence risk of HCC according to the random-effects model.

**FIGURE 3:**
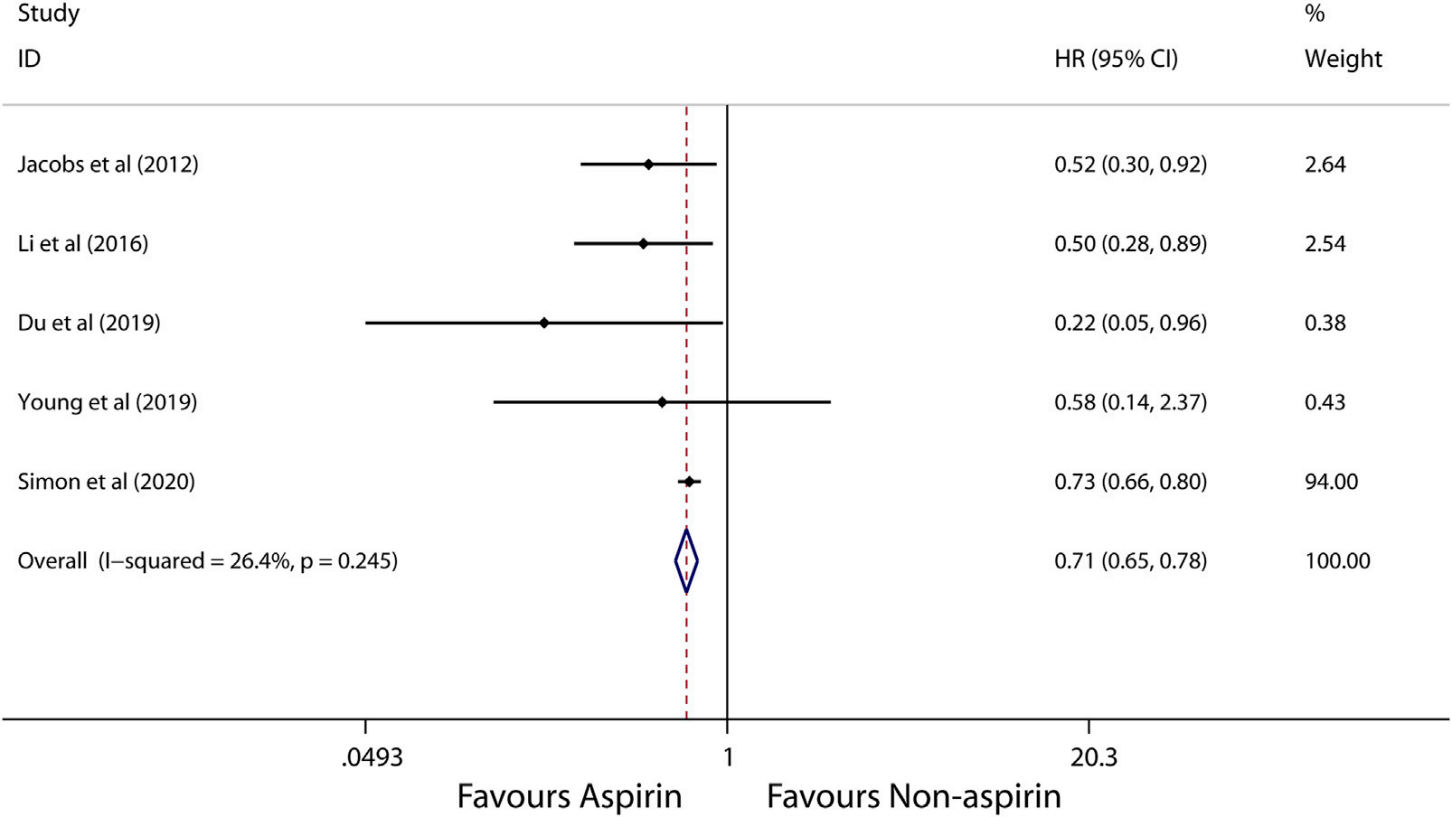
Meta-analysis of overall pooled HRs with 95% CIs across studies for secondary outcomes. Forest plot showing the significance of the relationship between aspirin use and mortality of HCC according to the fixed-effects model.

**FIGURE 4:**
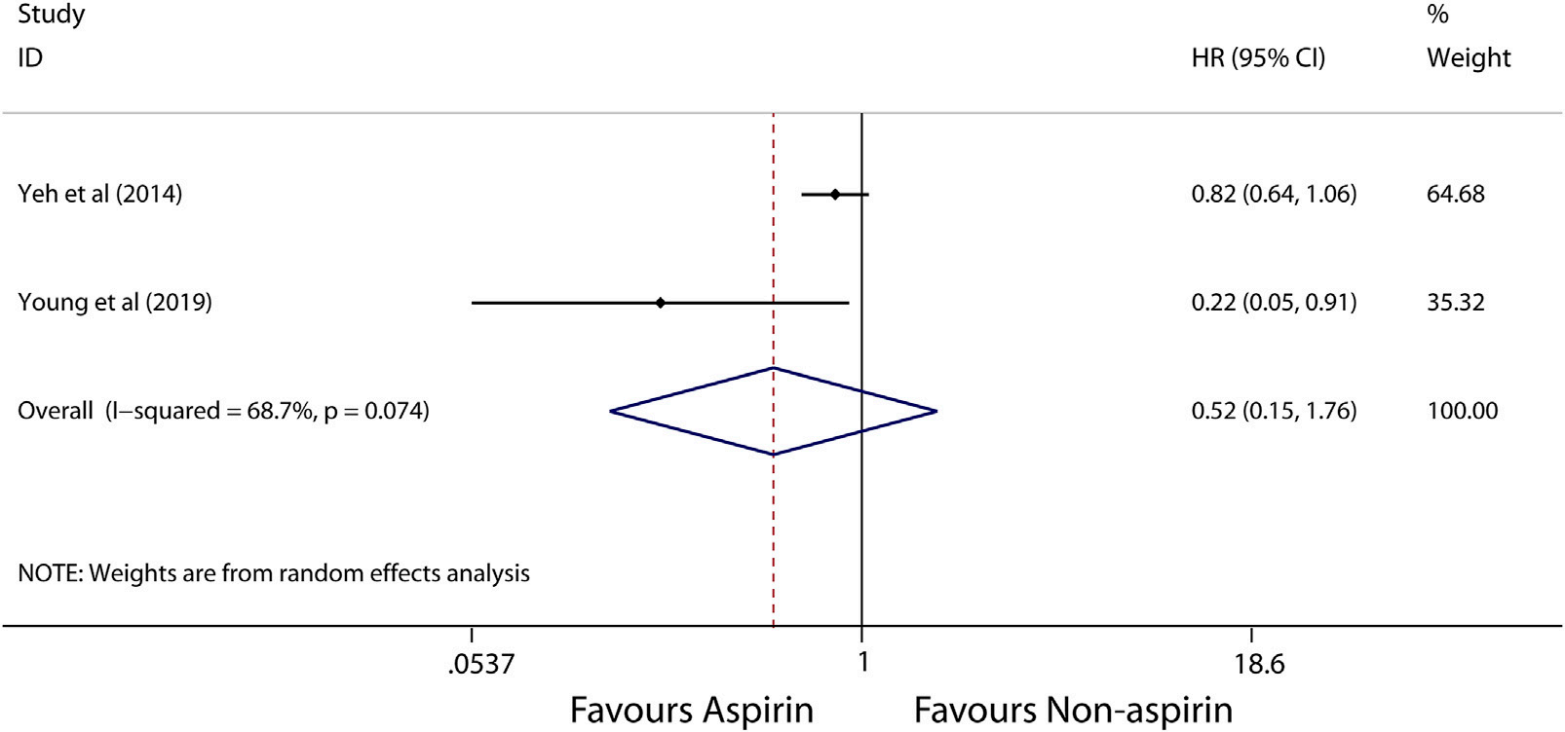
Meta-analysis of overall pooled HRs with 95% CIs across studies for secondary outcomes. Forest plot showing the significance of the relationship between aspirin use and recurrence of HCC according to the random-effects model.

A random-effects model was used to perform in this meta-analysis for incidence and recurrence due to substantial heterogeneity (*I* ^2^ = 92.5%, 68.7%, respectively), and fixed-effects model was used to perform in this meta-analysis for mortality due to mild heterogeneity (*I* ^2^ = 26.4%) between studies. The pooled results from these studies showed that aspirin is associated with lower incidence of HCC (HR, 0.56; 95% CI, 0.46-0.69, *p* < 0.001; *I* ^2^ = 92.5%; Figure 2). These articles suggest that aspirin is associated with a lower risk of HCC mortality (HR, 0.71; 95% CI, 0.65-0.78; *p* < 0.001; *I* ^2^ = 26.4%; Figure 3), not HCC recurrence (HR, 0.52; 95% CI, 0.15-1.76; *p* = 0.291; *I* ^2^ = 68.7%; Figure 4).

### 3.4 Subgroup Analysis

**FIGURE 5:**
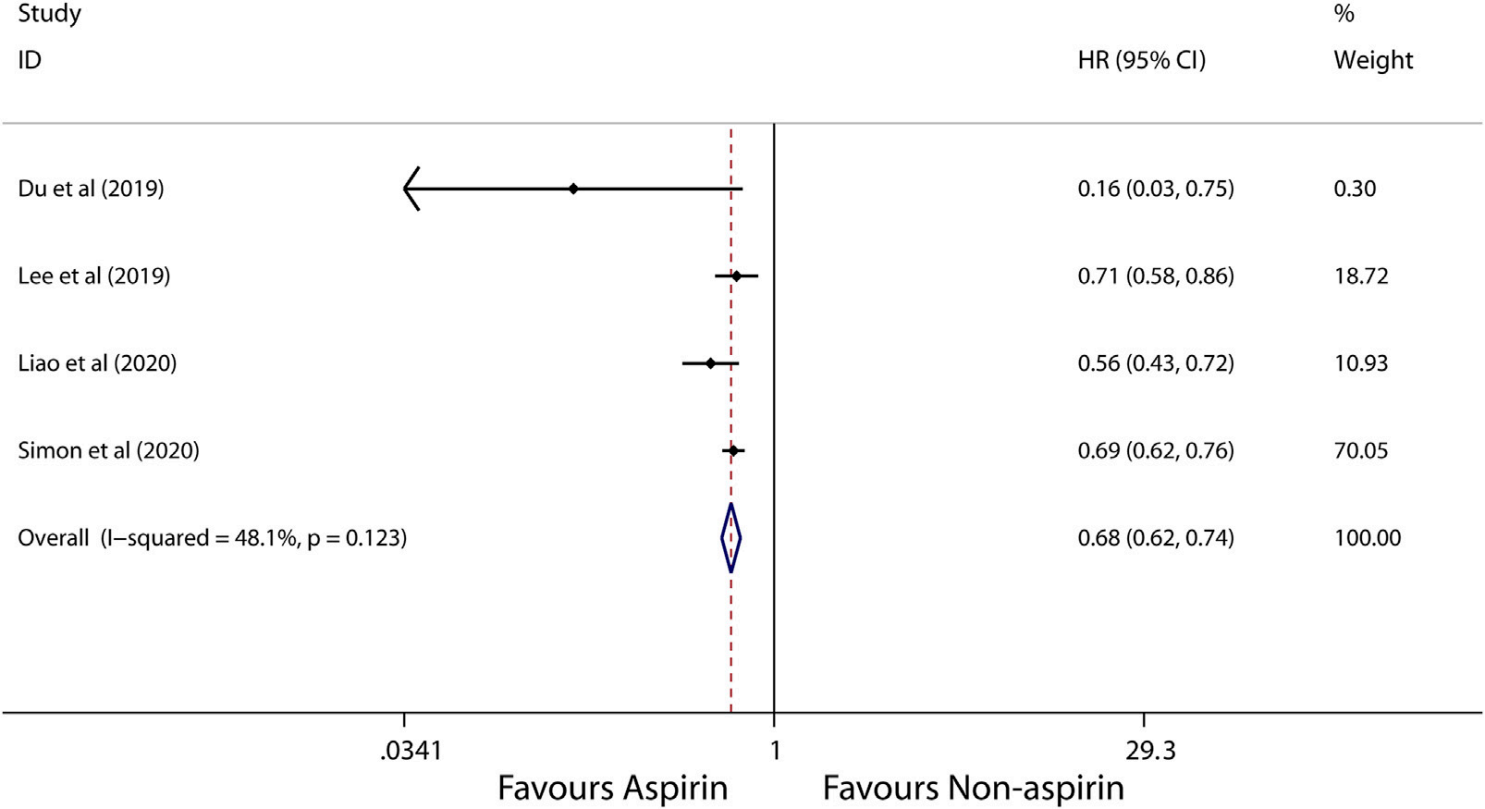
Meta-analysis of overall pooled HRs with 95% CIs across studies for primary outcomes in subgroup analyses. Forest plot showing the significance of the relationship between aspirin use and incidence risk of HCC in alcoholic cirrhosis patients according to the fixed-effects model.

**FIGURE 6:**
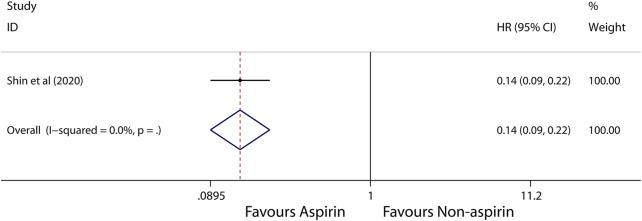
Meta-analysis of overall pooled HRs with 95% CIs across studies for primary outcomes in subgroup analyses. Forest plot showing the significance of the relationship between aspirin use and incidence risk of HCC in virus hepatitis patients according to the fixed-effects model.

Subgroup analysis further showed that use of aspirin is linked to a lower incidence of HCC in patients with alcoholic cirrhosis (HR, 0.14; 95% CI, 0.09-0.22; *p* < 0.001; *I* ^2^ = 0%; Figure 5), virus hepatitis (HR, 0.68; 95% CI, 0.62-0.74; *p* < 0.001; *I* ^2^ = 48.1%; Figure 6). Obviously, aspirin is associated with a lower incidence rate of liver cancer in alcoholic cirrhosis.

### 3.5 Sensitivity Analyses

Since the included studies were observational studies, the risk of bias was low (Supplementary Table S3), we did not conduct a sensitivity analysis of the methodological criteria. So we conduct a sensitivity analysis to evaluate the effect of any one study on the pooled HRs and 95% CIs by removing one individual study at a time. The results showed that the meta-analysis is robust and reliable (Supplementary Figure S1).

### 3.6 Publication Bias

We employed the funnel plots (Supplementary Figure S2) and the egger’s regression asymmetry tests (Supplementary Figure S3) to evaluate potential publication bias of the included studies. We observed a slight asymmetry in the funnel plots and the Egger’s Publication bias plot. However the results of Egger’s regression asymmetry tests confirmed no publication bias exist in these studies (*p* = 0.594).

## 4 Discussion

Evaluation of studies that enrolled 2781100 participants demonstrated that the incidence of HCC was lower in patients with alcoholic cirrhosis and virus hepatitis who use aspirin than that of patients who do not use aspirin, but also the mortality of HCC was lower in aspirin users than those in non-aspirin users. This finding suggests that the use of aspirin was associated with decreased risk of HCC and the risk of HCC mortality. However, clinical trials should be further conducted to confirm this suggestion.

Aspirin is a first-line nonsteroidal anti-inflammatory drug (NSAID), which has been reported to have chemo-protective effects, according to epidemiological researches. Studies ((Gann et al., 1993; Cook et al., 2005; Flossmann and Rothwell 2007; Burn et al., 2011)), in which patients with different genders and are from different regions were enrolled, have indicated the association between aspirin and cancer risk. While some recent studies have reported that NSAID ((Sahasrabuddhe et al., 2012; Petrick et al., 2015)), especially for aspirin, may be able to protect against incidence risk of HCC, other studies have indicated that non-aspirin NSAID could also reduce the number of HCC incidence ((Pang et al., 2017; Tao et al., 2018)). Thus, to further clarify this, we evaluated several studies ((Pang et al., 2017; Tao et al., 2018), (Simon et al., 2018; Liao et al., 2020; Shin et al., 2020), (Oh et al., 2017)), in which aspirin users and non-aspirin users were compared. Our investigation suggested that aspirin was associated with a reduced risk of HCC. And the subgroup analysis result of our meta-analysis also indicated that aspirin use was associated with a reduced incidence of HCC in virus hepatitis and alcoholic cirrhosis patients. Similar effects or mechanisms have also been observed in chronic hepatitis patients ((Maini and Schurich 2012; Sitia et al., 2013)). The presence of hepatitis virus causes CD8^+^ lymphocytes to secret many inflammatory factors involving in the dealing with infection; inability to timely clear the virus can lead to failure of the liver. Although numerous articles have demonstrated that aspirin users has a significantly lower incidence of HCC compared with non-aspirin users ((Maini and Schurich 2012; Sitia et al., 2013), (Maini and Schurich 2012; Sitia et al., 2013), (Maini and Schurich 2012; Sitia et al., 2013), (Maini and Schurich 2012; Sitia et al., 2013), (Simon et al., 2019), (Lee et al., 2017)), contradictory results have been reported by some studies ((Chiu et al., 2011; Kim et al., 2017), (Chiu et al., 2011; Kim et al., 2017)). Moreover, a meta-analysis that included 5 studies for incidence and 2 studies for mortality reported aspirin use could reduce the incidence risk of HCC((Chiu et al., 2011; Kim et al., 2017)), and also the 2-years and 4-years mortalities in patients with HCC. And the result is consistent with our meta-analysis. However another meta-analysis that included 5 studies reported aspirin use could not reduce the incidence risk of HCC((Chiu et al., 2011; Kim et al., 2017)). In addition, A recent meta-analysis included 8 cohort studies by Wang et al. focused on the dose-response effect of aspirin use and incidence risk of HCC((Chiu et al., 2011; Kim et al., 2017)), and which reported that the higher the aspirin dose, the lower incidence risk of HCC. However, all these three previous meta-analysis may lead to the in-reliable result as their small number of included studies ((Pang et al., 2017; Tao et al., 2018; Wang et al., 2020)). Besides, we also look forward to more studies on the dose dependence or time dependence between aspirin and HCC occur. In all, the majority of evidences shown above indicate that use of aspirin could reduce the incidence risk of HCC, also aspirin use and lower mortality in HCC patients.

Several mechanisms may support the beneficial effects of aspirin on the incidence risk of HCC. Aspirin has a very short half-life in the serum; it selectively inhibits the platelet COX-1. Patients with inflammation-related cancer, including HCC, have increased levels of pro-inflammatory factor, COX-2 enzyme ((Lim et al., 2000; Yip-Schneider et al., 2000; Singh et al., 2005; Chan et al., 2007)). Animal studies have suggested that aspirin at high doses is required to completely inhibit COX-2 ((Kern et al., 2002; Foderà et al., 2004; Chen et al., 2017)). Additionally, higher COX-2 levels can induce the hepatocarcinogenesis-related inflammatory factor cascades, such as protein kinase 3 (PK-3) and nuclear factor κB (NF-κB) pathways ((Iannacone et al., 2005)). Aspirin at high doses could block the NF-κB and PK-3 pathways ((Chan et al., 1998; Leng et al., 2003; Patrono et al., 2005)). The mechanisms in which use of aspirin may benefit HCC patients with HBV are as follows: while platelets accelerate HBV-related liver injury by retaining inflammation (69), aspirin exerts its anti-inflammation by inhibiting the production of thromboxane A2 and relevant platelet activation pathways ((Cattaneo 2004; Aiolfi and Sitia 2015)). Moreover, the use of aspirin antiplatelet therapy reduces the frequency of platelet immune cell interaction and intrahepatic platelet accumulation, thereby limiting the transport of hepatic immune cells, which can prevent the development of non-alcoholic steatohepatitis (NASH) and subsequent HCC((Malehmir et al., 2019)). Other studies have suggested that anti-platelet therapy can diminish not only the intrahepatic HBV-specific CD8 T cells ((Cattaneo 2004; Aiolfi and Sitia 2015)), but also normal inflammatory cells; as a result, HCC is developing in the construction of HBV transgenic animal model. A study of a xenograft nude mouse model have also indicated that use of aspirin could inhibit growth and/or death of HCC tumor ((Cattaneo 2004; Aiolfi and Sitia 2015), (Li et al., 2013b)).

This study has several advantages. First, the total sample sizes are sufficient for this meta-analysis; therefore, the bias from sample sizes can be diminished; Second, it is the first systematic review and meta-analysis to analyses the incidence of HCC in aspirin users in patients with virus hepatitis and alcoholic cirrhosis. Third, the results shown in the NOS quality list showed that this article may have a risk of bias, suggesting the methodology used in the original studies has higher quality; Moreover, the outcome data in the included studies were adjusted into the primary and secondary outcomes; hence, analyses of effects of aspirin on the incidence, and recurrence and mortality of HCC were conducted separately. Furthermore, the synthetic HR and its 95% CI were obtained using the generic inverse variance methods of random-effect models; thus, the obtained HR indicates the sum effect of aspirin on HCC incidence. More importantly, although all the included studies are observational studies, the main confounding variables that impacted the primary results that were adjusted in most of original articles. Finally the sensitivity analysis results suggested that the meta-analysis employed in this study is robust and reliable.

Despite the above strengths, this study has some limitation: First, high heterogeneity was observed in the sum effect of aspirin on the incidence of HCC, suggesting the results presented here may be different from those in the original articles (NOS assessment, however, indicated that the conclusions presented here is reliable); Second, aspirin dose and period of use were varied in the original studies, which may affect the results shown in this study and may be the source of heterogeneity; therefore, further researches are needed; Third, the small included studies for aspirin use and incidence of HCC in alcoholic cirrhosis patients may lead to the in-reliable result. However, it is clear that use of aspirin has a significant reduction in the incidence of HCC in patients with alcoholic cirrhosis and virus hepatitis. Fourth, this study evaluate only the preventive or therapeutic effect of aspirin on HCC patients, not its safety, administration initiation or continuation; Finally, more importantly, although the included articles have a low risk of bias, this study did not include randomized controlled trials, which may be one of the most important disadvantages.

This article presents the first meta-analysis that elaborate on the beneficial effect of aspirin on HCC incidence in patients with or without virus hepatitis and alcoholic cirrhosis. The analysis indicated that aspirin could decrease the incidence risk of HCC including virus hepatitis, especially for patients with alcoholic cirrhosis, and also the mortality of HCC. However, further clinical trials are encouraged to be conducted to support the present results.

## Data Availability

The original contributions presented in the study are included in the article/Supplementary Material, further inquiries can be directed to the corresponding authors.
