# Supplementary figures and images for: Systematic Review and Meta-analysis: Association of Aspirin With Incidence of Hepatocellular Carcinoma
Source: Front Pharmacol. 2022 Mar 1;13:764854. doi: 10.3389/fphar.2022.764854 (PMC8921872; doi:10.3389/fphar.2022.764854)

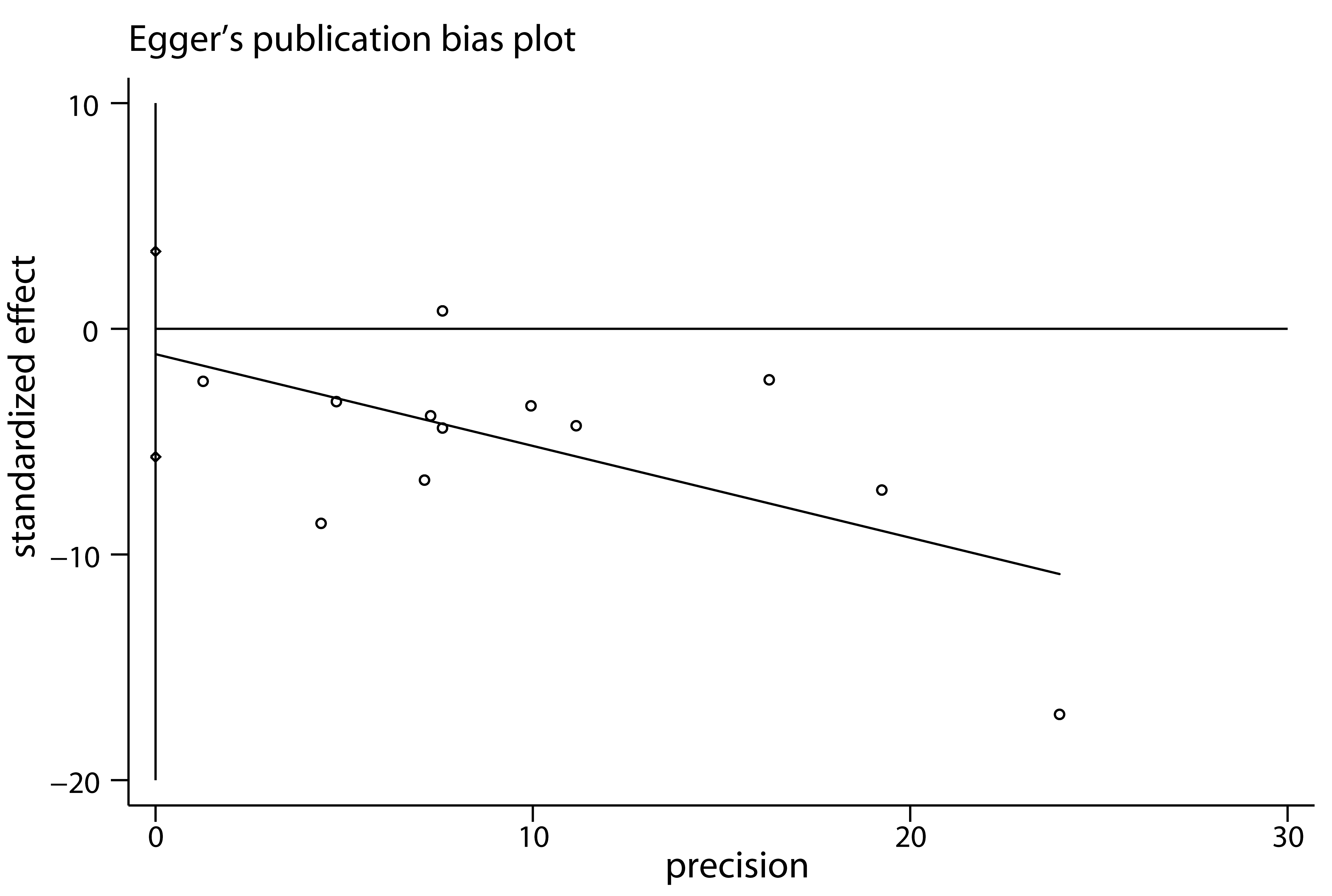

Supplement: Supplementary file 1 [file Image3.TIF]

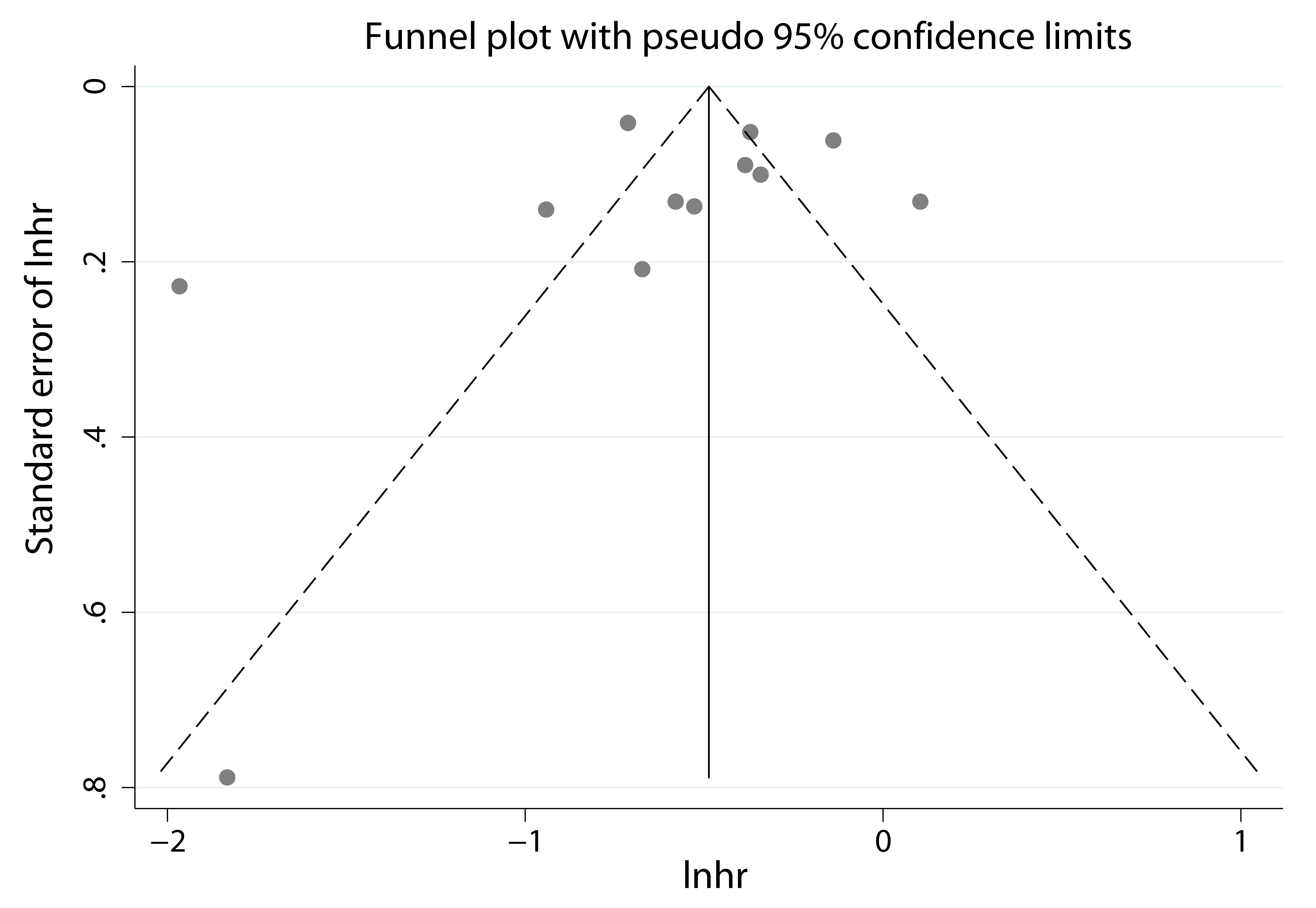

Supplement: Supplementary file 3 [file Image2.TIF]

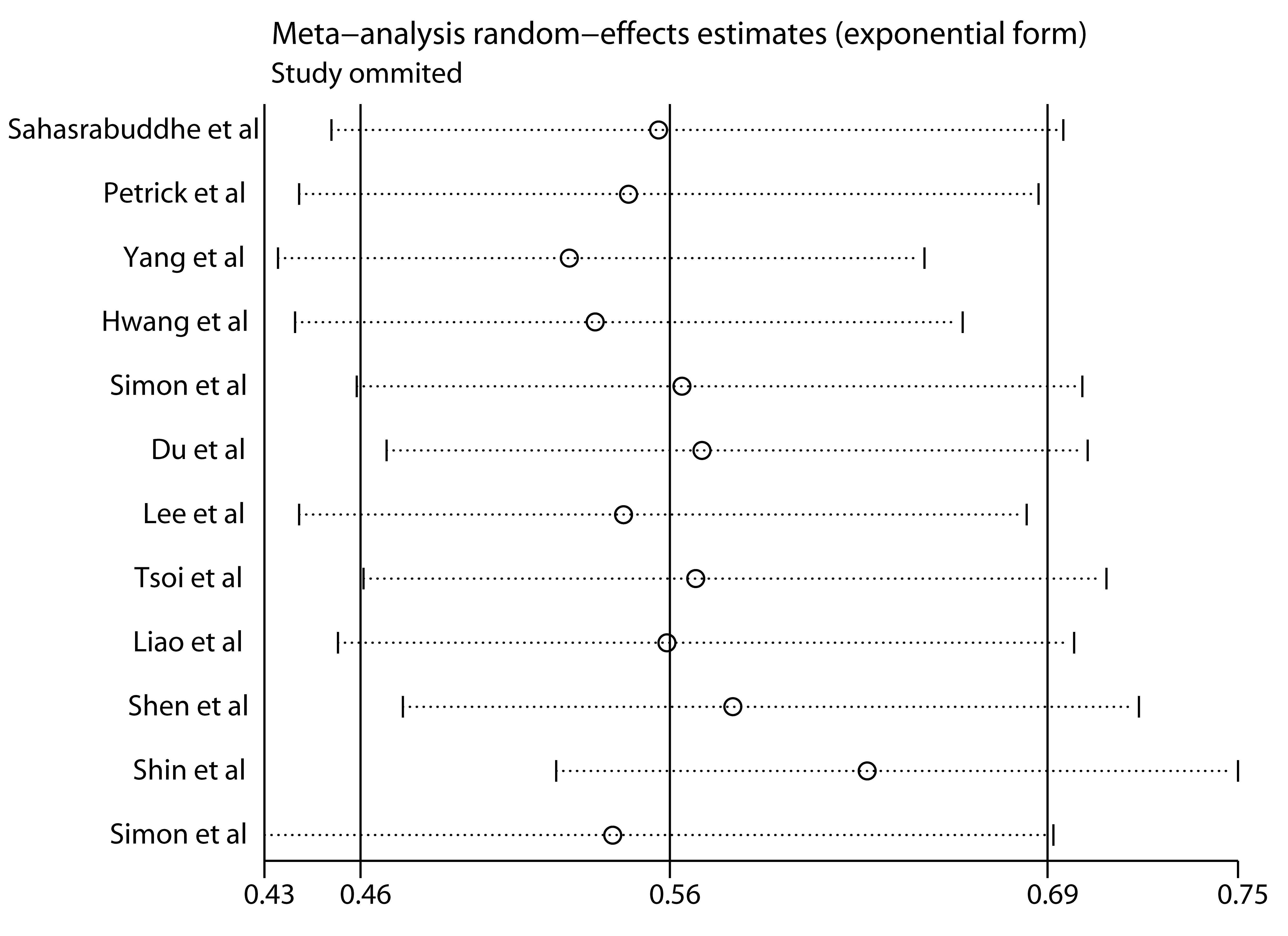

Supplement: Supplementary file 4 [file Image1.TIF]
