# Supplementary material for: Systematic Review and Meta-analysis: Association of Aspirin With Incidence of Hepatocellular Carcinoma
Source: Front Pharmacol. 2022 Mar 1;13:764854. doi: 10.3389/fphar.2022.764854 (PMC8921872; doi:10.3389/fphar.2022.764854)
Supplement: Supplementary file 2 [file Table3.doc]

| **Studies**  First Author |  | **Selection** | | |  | **Comparability** | | |  | **Assessment of outcome** | | |  | **Total Quality Score** |
| --- | --- | --- | --- | --- | --- | --- | --- | --- | --- | --- | --- | --- | --- | --- |
|  |  | Representativeness of aspirin Use Arm(s) | Selection  of the non-aspirin Use Arm(s) | Origin of Exposure Source |  | Demonstration that Outcome of Interest was not Present at Start of Study | Studies Controlling the Most Important Factors | Studies Controlling the Other Main Factors |  | Assessment of Outcome with Independency | Adequacy of Follow-up Length (to assess outcome) | Lost to Follow-up Acceptable (less than 10% and reported) |  |  |
| Jacobs et al (2012)[^41^](#_ENREF_41) |  | * | * | * |  | * | * |  |  | * | * |  |  | 7 |
| Sahasrabuddhe et al (2012)[^21^](#_ENREF_21) |  | * | * | * |  | * | * | * |  | * | * |  |  | 8 |
| Yeh et al (2014)[^44^](#_ENREF_44) |  | * | * | * |  | * | * | * |  | * | * |  |  | 8 |
| Petrick et al (2015)[^20^](#_ENREF_20) |  | * | * | * |  | * | * | * |  | * | * |  |  | 8 |
| Li et al (2016)[^42^](#_ENREF_42) |  | * | * | * |  | * | * | * |  | * | * |  |  | 8 |
| Yang et al (2016)[^22^](#_ENREF_22) |  | * | * | * |  | * | * | * |  | * | * |  |  | 8 |
| Hwang et al (2018)[^19^](#_ENREF_19) |  | * | * | * |  | * | * | * |  | * | * |  |  | 8 |
| Simon et al (2018) [^36^](#_ENREF_36) |  | * | * | * |  | * | * | * |  | * | * |  |  | 8 |
| Du et al (2019)[^40^](#_ENREF_40) |  | * | * | * |  | * | * | * |  | * | * |  |  | 8 |
| Lee et al (2019)[^34^](#_ENREF_34) |  | * | * | * |  | * | * |  |  | * | * |  |  | 7 |
| Tsoi et al (2019)[^35^](#_ENREF_35) |  | * | * | * |  | * | * |  |  | * | * |  |  | 7 |
| Young et al (2019)[^43^](#_ENREF_43) |  | * | * | * |  | * | * | * |  | * | * |  |  | 8 |
| Liao et al (2020)[^37^](#_ENREF_37) |  | * | * | * |  | * | * | * |  | * | * |  |  | 8 |
| Shen et al (2020)[^24^](#_ENREF_24) |  | * | * | * |  | * | * | * |  | * | * |  |  | 8 |
| Simon et al (2020)[^39^](#_ENREF_39) |  | * | * | * |  | * | * | * |  | * | * |  |  | 8 |
| Shin et al (2020)[^38^](#_ENREF_38) |  | * | * | * |  | * | * | * |  | * | * |  |  | 8 |

**Supplementary Table 3** The Newcastle-Ottawa Scale (NOS) for assessing the quality of including studies
