# Supplementary material for: Systematic Review and Meta-analysis: Association of Aspirin With Incidence of Hepatocellular Carcinoma
Source: Front Pharmacol. 2022 Mar 1;13:764854. doi: 10.3389/fphar.2022.764854 (PMC8921872; doi:10.3389/fphar.2022.764854)
Supplement: Supplementary file 6 [file Table2.docx]

**Supplementary Table 2. The detailed search strategy**

| **Electronic databases** | **Search** | **Search strategy** | **Results** |
| --- | --- | --- | --- |
| **MEDLINE** | #1 | ("Aspirin"[MeSH Terms]) OR "Aspirin"[Title/Abstract] | 66514 |
|  | #2 | ("acetylsalicylic acid"[MeSH Terms]) OR "acetylsalicylic acid"[Title/Abstract] | 9507 |
|  | #3 | #1 OR #2 | 69433 |
|  | #4 | ("carcinoma, hepatocellular"[MeSH Terms]) OR "hepatocellular carcinoma"[Title/Abstract] | 113877 |
|  | #5 | ("liver neoplasms"[MeSH Terms]) OR "liver neoplasms"[Title/Abstract] | 163700 |
|  | #6 | "liver cancer"[Title/Abstract] OR "hepatic cellular cancer"[Title/Abstract] OR "HCC"[Title/Abstract] | 67802 |
|  | #7 | #4 OR #5 OR #6 | 198257 |
|  | #8 | #3 AND #7 | **168** |
| **EMBASE** | #1 | 'aspirin'/de OR 'acetylsalicylic acid'/de | 211840 |
|  | #2 | 'aspirin':ti,ab,kw OR 'acetylsalicylic acid':ti,ab,kw | 84974 |
|  | #3 | #1 OR #2 | 221691 |
|  | #4 | 'hepatocellular carcinoma'/de OR 'liver cancer'/de | 185934 |
|  | #5 | 'hepatocellular carcinoma':ti,ab,kw OR 'liver cancer':ti,ab,kw OR 'hepatic cellular cancer':ti,ab,kw OR 'hcc':ti,ab,kw | 152488 |
|  | #6 | #4 OR #5 | 214133 |
|  | #7 | #3 AND #6 | **699** |
| **Cochrane CENTRAL** | #1 | MeSH descriptor: [Aspirin] explode all trees | 5781 |
|  | #2 | (Aspirin):ti,ab,kw | 13488 |
|  | #3 | (Acetylsalicylic acid):ti,ab,kw | 5176 |
|  | #4 | #1 OR #2 OR #3 | 15359 |
|  | #5 | MeSH descriptor: [Carcinoma, Hepatocellular] explode all trees | 1680 |
|  | #6 | MeSH descriptor: [Liver Neoplasms] explode all trees | 2838 |
|  | #7 | (Hepatocellular Carcinoma):ti,ab,kw | 4471 |
|  | #8 | (Liver cancer):ti,ab,kw | 9392 |
|  | #9 | (hepatic cellular cancer):ti,ab,kw | 48 |
|  | #10 | (HCC):ti,ab,kw | 3034 |
|  | #11 | #5 OR #6 OR #7 OR #8 OR #9 OR #10 | 12984 |
|  | #12 | #4 AND #11 | **48** |
| **Web of Science** | #1 | TOPIC: (Acetylsalicylic acid) OR TITLE: (Acetylsalicylic acid) | 3203 |
|  | #2 | TOPIC: (Aspirin) OR TITLE: (Aspirin) | 22416 |
|  | #3 | #1 OR #2 | 24114 |
|  | #4 | TOPIC: (Hepatocellular Carcinoma) OR TITLE: (Hepatocellular Carcinoma) | 38964 |
|  | #5 | TOPIC: (Liver cancer) OR TITLE: (Liver cancer) | 76204 |
|  | #6 | TOPIC: (hepatic cellular cancer) OR TITLE: (hepatic cellular cancer) | 1336 |
|  | #7 | TOPIC: (HCC) OR TITLE: (HCC) | 41637 |
|  | #8 | #4 OR #5 OR #6 OR #7 | 116641 |
|  | #9 | #3 AND #8 | **183** |
